# The Chemical Bond: When Atom Size Instead of Electronegativity Difference Determines Trend in Bond Strength

**DOI:** 10.1002/chem.202103544

**Published:** 2021-10-19

**Authors:** Eva Blokker, Xiaobo Sun, Jordi Poater, J. Martijn van der Schuur, Trevor A. Hamlin, F. Matthias Bickelhaupt

**Affiliations:** ^1^ Department of Theoretical Chemistry Amsterdam Institute of Molecular and Life Sciences (AIMMS) Amsterdam Center for Multiscale Modeling (ACMM) Vrije Universiteit Amsterdam De Boelelaan 1083 1081 HV Amsterdam The Netherlands; ^2^ Departament de Química Inorgànica i Orgànica & IQTCUB Universitat de Barcelona Martí i Franquès 1–11 08028 Barcelona Spain; ^3^ ICREA Pg. Lluís Companys 23 08010 Barcelona Spain; ^4^ Polymer Specialties, Nouryon Zutphenseweg 10 7418 AJ Deventer The Netherlands; ^5^ Institute of Molecules and Materials Radboud University Heyendaalseweg 135 6525 AJ Nijmegen The Netherlands

**Keywords:** Bond energy, Bond theory, Density functional calculations, Main group elements, Thermochemistry

## Abstract

We have quantum chemically analyzed element−element bonds of archetypal H_n_X−YH_n_ molecules (X, Y=C, N, O, F, Si, P, S, Cl, Br, I), using density functional theory. One purpose is to obtain a set of consistent homolytic bond dissociation energies (BDE) for establishing accurate trends across the periodic table. The main objective is to elucidate the underlying physical factors behind these chemical bonding trends. On one hand, we confirm that, along a period (e. g., from C−C to C−F), bonds strengthen because the electronegativity difference across the bond increases. But, down a period, our findings constitute a paradigm shift. From C−F to C−I, for example, bonds do become weaker, however, not because of the decreasing electronegativity difference. Instead, we show that the effective atom size (via steric Pauli repulsion) is the causal factor behind bond weakening in this series, and behind the weakening in orbital interactions at the equilibrium distance. We discuss the actual bonding mechanism and the importance of analyzing this mechanism as a function of the bond distance.

## Graphical abstract

**Paradigm Switch**! Chemical bonds in general become stronger as the electronegativity difference across the bond increases. This correlation has hitherto been considered causal but, here, we show it is not in some remarkable cases. For example, carbon−halogen bonds become weaker from C−F to C−I, despite stronger (not weaker) orbital interactions as the bond overlap increases, because of stronger Pauli repulsion with the larger, more electron‐rich halogen.

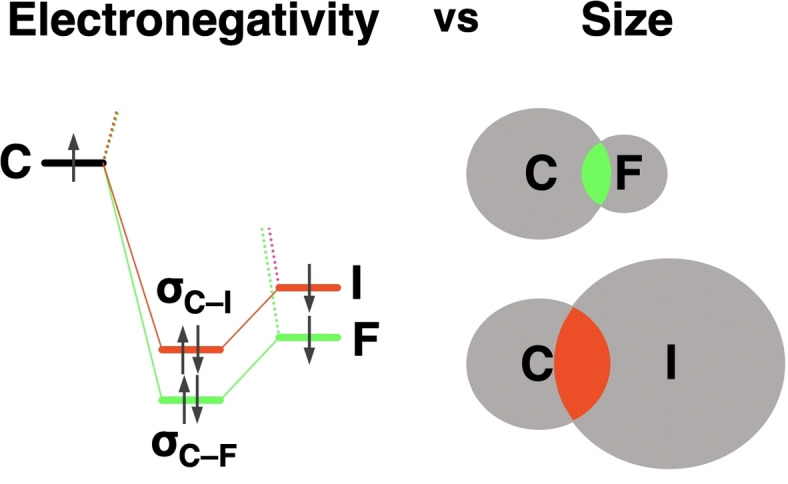

The chemical bond is a key concept in chemistry.[^1^, ^2^, ^3^, ^4^] Structure, stability and reactivity of molecules critically depend on the length and, especially, the stability of chemical bonds. A sound and minute understanding of trends in element−element bond strengths across the periodic table is, therefore, indispensable for chemical theory and rational design in the molecular sciences. A well‐known example of such a trend is that a more polar bond X−Y often tends to be stronger than a related but less polar bond, as reflected by the bond dissociation enthalpy (BDE; see Equation 1).[^1^, ^2^, ^3^, ^4^, ^5^]

(1) $$X-Y\to X{}^{•}+Y{}^{•}\;\Delta H=\mathrm{BDE}$$

The accepted picture behind this trend is that the larger electronegativity difference across the X−Y bond leads to a greater stabilization of the bonding electron stemming from the more electropositive radical fragment. From a molecular orbital (MO) perspective, this is understood as the more stabilizing orbital interaction as the electron of the higher‐energy singly‐occupied molecular orbital (SOMO) drops deeper in energy into the bonding combination with the lower‐energy SOMO in the case of a larger orbital‐energy gap (*vide infra*).^[3]^ A textbook example is the weakening of the carbon−halogen bond in H_3_C−Y along Y=F, Cl, Br and I.^[4]^ Despite a number of bonding studies on first‐ and second‐row elements,^[6]^ and other studies into the chemical bond,^[7]^ little quantitative knowledge of the actual bonding mechanism of polar bonds exists beyond the arguments based on electronegativity differences.[^8^, ^9^, ^10^, ^11^, ^12^, ^13^, ^14^, ^15^]

Herein, we show based on detailed quantum chemical analyses how, and why, the electronegativity model for the strength of polar bonds breaks down for certain series (C−F to C−I) whereas it holds for others (C−C to C−F). Interestingly, the series of carbon‐halogen bonds, for which the electronegativity model breaks down, has hitherto served to illustrate this textbook model.[^4^, ^10^, ^16^, ^17^]

Thus, we have explored and analyzed the length and strength (BDE) of single bonds X−Y derived from elements across the periodic table (X, Y=periods 2–3, groups 14–17, and Br, I) using dispersion‐corrected density functional theory (DFT) and quantitative canonical MO theory in conjunction with a matching bond energy decomposition analysis (EDA) using ADF.[^18^, ^19^, ^20^] Not only do we provide accurate trends in BDEs for all possible X−Y electron pair bonds along model systems H_n_X−YH_n_, all consistently obtained at BLYP‐D3(BJ)/TZ2P,^[21]^ and for Br and I including ZORA;^[22]^ we also reveal the physical factors at play behind the computed trends, as already alluded to above. Interestingly, our explorations highlight the importance of carrying out bonding analyses as a function of the X−Y bond distance if one wishes to go beyond correlations and uncover the actual causalities in the bonding mechanism.

Table 1 provides all our computed H_n_X−YH_n_ bond dissociation enthalpies Δ*H* (BDE),^[23]^ using standard conditions (298.15 K and 1 atm) and the ideal gas model for thermodynamic corrections. The spectrum of BDEs in our model systems reaches from 48.0 kcal mol^−1^ for F−F till 148.6 kcal mol^−1^ for the strongest polar bond, H_3_Si−F. Furthermore, the BDE increases from C−C to C−F (85.2 to 111.3 kcal mol^−1^), and it decreases from C−F to C−Cl (111.3 to 80.9 kcal mol^−1^). Clearly, bond strengths correlate with the electronegativity difference Δχ=|χ_X_−χ_Y_| across the X−Y bond. This becomes even more obvious upon plotting BDEs as a function of the Pauling electronegativity χ of the main‐group elements, X and Y, involved in the X−Y bonds,^[8]^ in Figure 1. In some cases, such as, from C−C to C−N (85.2 to 80.3 kcal mol^−1^, see also Table 1), the simple trend of stronger BDE for larger Δχ is disturbed,^[24]^ however, by and large it holds (*vide infra*). The question, now, is whether these correlations along periods and groups are causal, or not.

**Table 1:** Bond dissociation enthalpies Δ*H* (BDE) of the H_n_X−YH_n_ systems.^[a]^

	YH_n_^.^							
H_n_X^.^	CH_3_^.^	NH_2_^.^	OH^.^	F^.^	SiH_3_^.^	PH_2_^.^	SH^.^	Cl^.^
H_3_C^.^	85.2	80.3	89.2	111.3	83.2	67.3	70.7	80.9
H_2_N^.^	80.3	60.7	59.8	74.2	96.7	68.3	63.9	60.8
HO^.^	89.2	59.8	52.9	55.9	118.1	86.5	69.5	57.8
F^.^	111.3	74.2	55.9	48.0	148.6	112.0	86.2	66.8
H_3_Si^.^	83.2	96.7	118.1	148.6	71.4	66.4	82.3	103.6
H_2_P^.^	67.3	68.3	86.5	112.0	66.4	54.4	63.3	75.9
HS^.^	70.7	63.9	69.5	86.2	82.3	63.3	61.7	63.8
Cl^.^	80.9	60.8	57.8	66.8	103.6	75.9	63.8	59.2

[a] Computed at BLYP‐D3(BJ)/TZ2P at 298.15 K and 1 atm.

**Figure 1:**
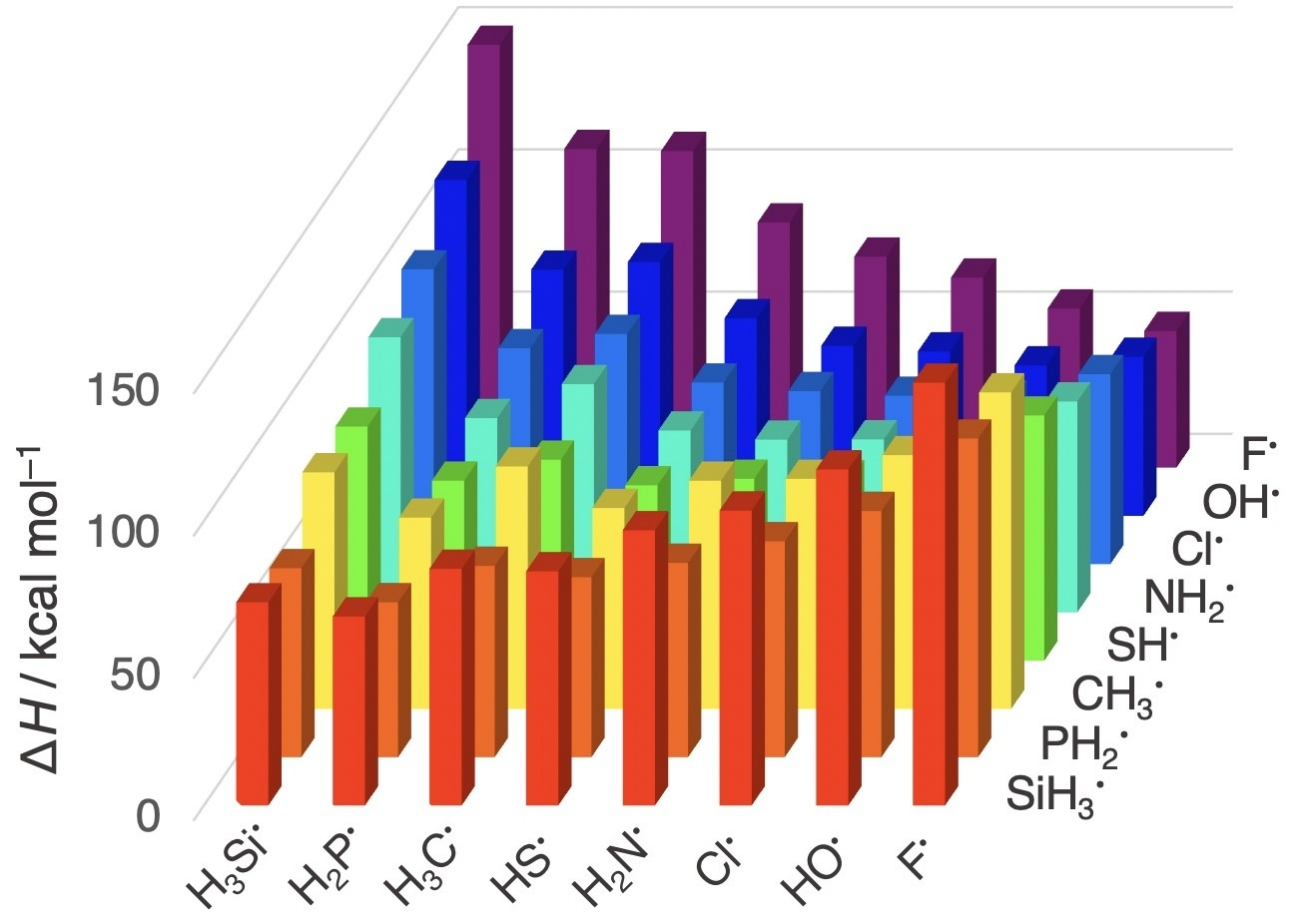
Bond dissociation enthalpy (BDE, in kcal mol^−1^) of the H_n_X−YH_n_ systems as a function of the Pauling electronegativity of the main‐group element [Pauling electronegativity χ from lowest to highest value: Si (1.90), P (2.19), C (2.55), S (2.58), N (3.04), Cl (3.16), O (3.44) and F (3.98)].^[8]^ BDE computed at BLYP‐D3(BJ)/TZ2P at 298.15 K and 1 atm.

Table S1 in the Supporting Information shows that the trends in Δ*H* are set by the electronic bond dissociation energies Δ*E*.[^5^, ^25^] We analyze the bond energy Δ*E* associated with the bond formation process X^.^+Y^.^→X−Y using the activation strain model in which Δ*E* is decomposed into the strain energy Δ*E*_strain_ and the interaction energy Δ*E*_int_.^[19a]^ The interaction energy Δ*E*_int_ can be further decomposed using our EDA method (see Table 2 for H_3_C−CH_3_, H_3_C−F, and H_3_C−Cl), into the classical electrostatic interaction Δ*V*_elstat_, the Pauli repulsion Δ*E*_Pauli_ (the destabilizing interaction between occupied orbitals), the orbital interaction Δ*E*_oi_ (accounts for electron‐pair bonding by the SOMO−SOMO interaction, charge transfer, and polarization), the dispersion energy Δ*E*_disp_, and the spin polarization Δ*E*_spinpol_.^[19a]^ Full details for all H_n_X−YH_n_ systems can be found in the Supporting Information, including an activation strain analysis, EDA, and a KS‐MO analysis as function of the bond distance for the combinations of CH_3_^.^, F^.^, SiH_3_^.^ and Cl^.^ (Figures S1–S3).

**Table 2:** H_3_C−CH_3_, H_3_C−F and H_3_C−Cl bonding mechanisms at the equilibrium and at a consistent geometry (in Å, kcal mol^−1^, eV) with the SOMO−SOMO gap Δ*ϵ* and overlap S.^[a,b]^

	*d*_X−Y_	Δ*E*	Δ*E*_strain_	Δ*E*_int_	Δ*V*_elstat_	Δ*E*_Pauli_	Δ*E*_oi_	Δ*ϵ*	S
Equilibrium geometry									
H_3_C−CH_3_	1.538	−92.1	18.4	−110.4	−129.5	204.6	−186.4	0.00	0.42
H_3_C−F	1.413	−115.3	6.3	−121.6	−105.3	254.0	−272.5	7.44	0.26
H_3_C−Cl	1.820	−84.2	5.9	−90.0	−96.1	172.8	−167.4	3.88	0.34
Consistent geometry									
H_3_C−CH_3_	1.400	−85.5	23.0	−108.4	−169.8	289.6	−229.1	0.00	0.42
H_3_C−F	1.400	−115.2	6.6	−121.8	−108.9	264.9	−280.1	7.42	0.26
H_3_C−Cl	1.400	−13.3	17.5	−30.8	−248.8	567.6	−350.3	3.16	0.35

[a] Computed at BLYP‐D3(BJ)/TZ2P. [b] The dispersion energy Δ*E*_disp_ (around −1.0 kcal mol^−1^) and the spin polarization Δ*E*_spinpol_ (around +2.5 kcal mol^−1^) are not shown.

The answer to our question, as revealed by our bonding analyses, is: No, along certain series of X−Y bonds, such as the carbon−halogen bonds (C−F to C−Cl), the correlation between BDE and Δχ is not causal but instead a side product of a different underlying mechanism. Along other series, such as the carbon‐second‐period‐element bonds (C−C to C−F), the correlation is in fact confirmed to be causal. In the following, we guide the reader through our analyses to see how and why the electronegativity model breaks down in certain cases, strikingly, in those cases that are generally used to illustrate its validity, the carbon−halogen bonds.^[4]^

First, we examine the carbon−halogen bonds by comparing C−F and C−Cl in Table 2. The strain energy Δ*E*_strain_, which results from the pyramidalization of the methyl fragment,^[26]^ is small (6.3 and 5.9 kcal mol^−1^), and, therefore, the bond weakening Δ*E* from −115.3 to −84.2 kcal mol^−1^ is determined by Δ*E*_int_ that becomes less stable from −121.6 to −90.0 kcal mol^−1^. The orbital interaction Δ*E*_oi_ that destabilizes from −272.5 to −167.4 kcal mol^−1^ seems the causal factor, following the decrease in SOMO−SOMO gap Δ*ϵ* (7.44 to 3.88 eV), i. e., the decrease in electronegativity difference. Figures 2a and 2b show schematic representations of a SOMO−SOMO interaction. For H_3_C−F, the low‐lying 2p_σ_ SOMO on the halogen engages in a 2‐center 3‐electron interaction with the filled σ_C−H_ orbitals, which pushes up the σ‐bonding orbital but effectively this does not alter the trends (Figure 2b). Therefore, we could distill the orbital interaction scheme from C−F to C−Cl to Figure 2c, where the magnitude of the energy gap Δ*ϵ* determines Δ*E*_oi_, and thus the bond strength.

**Figure 2:**
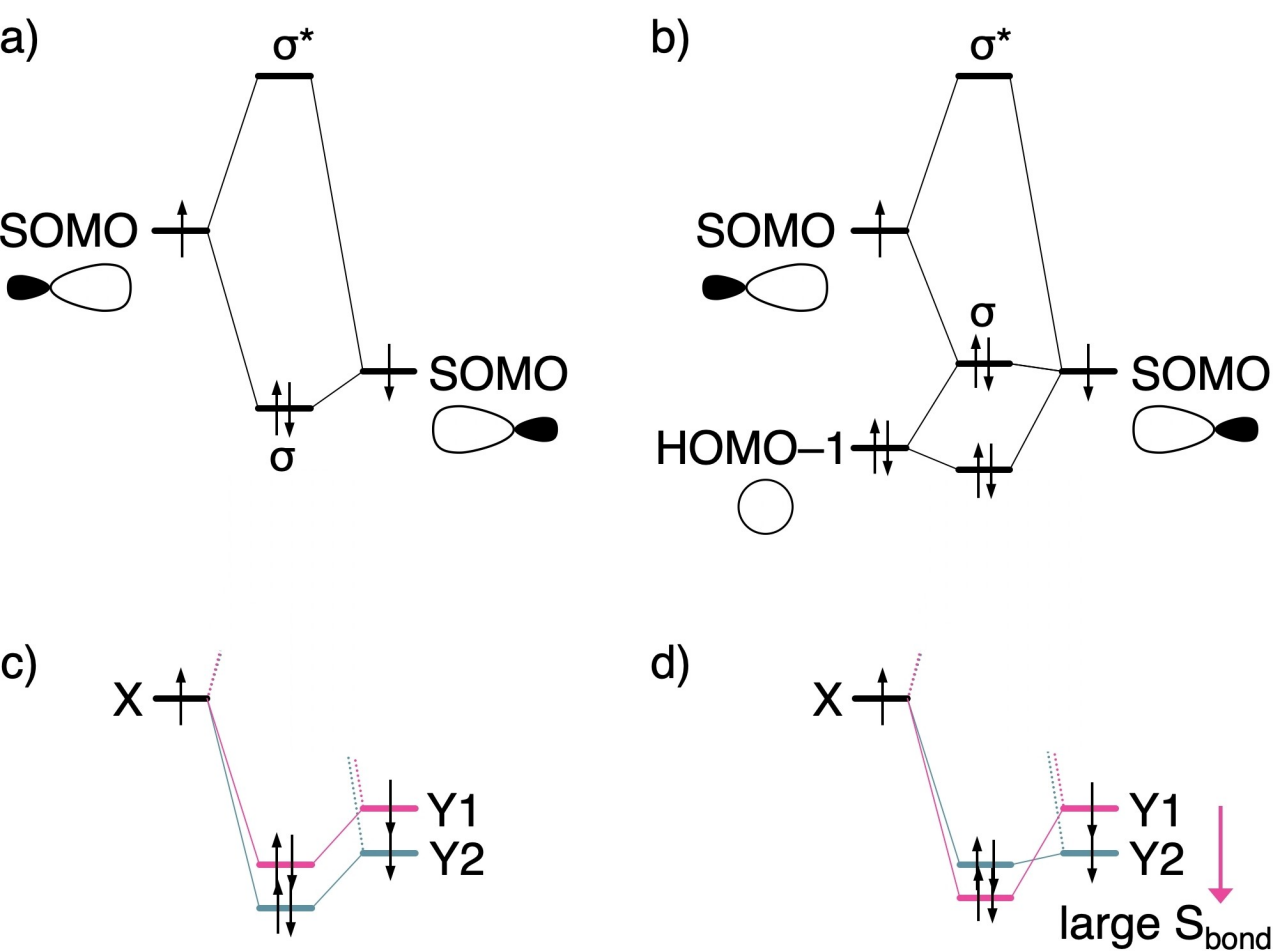
Schematic orbital interaction diagrams: a) SOMO−SOMO interaction; b) SOMO−SOMO interaction in the presence of a lower‐lying occupied orbital; c) X−Y bond with radical Y1 leading to a smaller Δ*E*_oi_ stabilization; and d) radical Y1 leading to a larger Δ*E*_oi_ stabilization.

Intriguingly, however, the bond weakening is not caused by Δ*E*_oi,_ since, at any given bond distance, the latter is more stabilizing for C−Cl than for C−F (blue versus green striped lines in Figure 3a). The reason for this unexpected order in stabilization is a substantially better overlap S (Figure 3c, solid lines) of the comparatively diffuse CH_3_ SOMO with the valence np_σ_ orbital of the heavier, and also more diffuse halogen Cl (Figure 4). The larger, more favorable SOMO−SOMO overlap for the C−Cl bond thus overrules the unfavorable decrease in energy gap (Figure 3c, dashed lines). We depict this schematically in Figure 2d, where the interaction with the smaller energy gap has now the largest Δ*E*_oi_ stabilization (the X−Y1 bond) due to a larger bond overlap S_bond_. Our analysis as a function of the bond distance reveals that the electronegativity model cannot be the reason for the stronger bond for C−F than for C−Cl as suggested in authoritative textbooks, for example, by Anslyn.^[4]^

**Figure 3:**
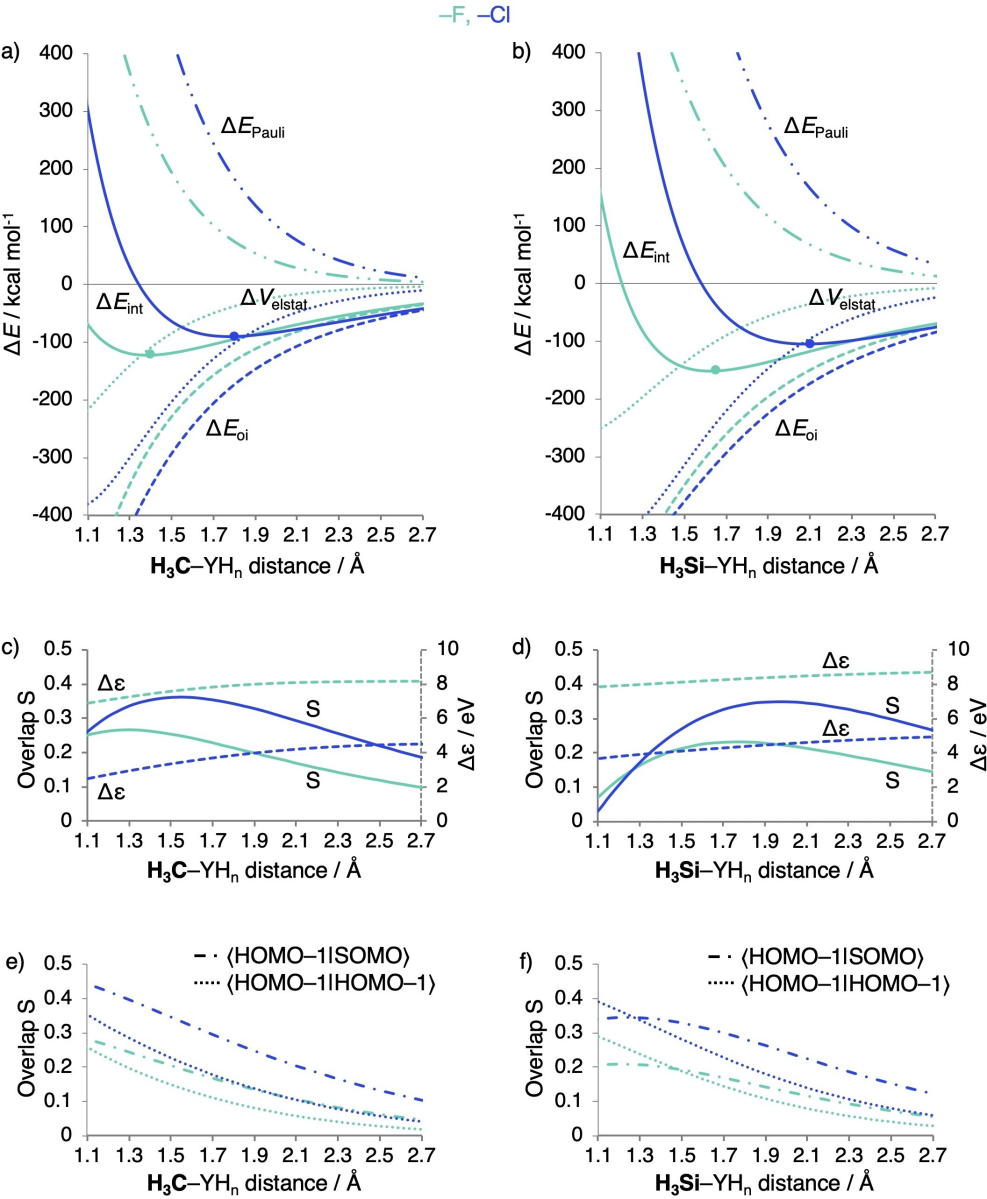
a–b) Energy decomposition analysis, c–d) SOMO−SOMO overlap S $\left\langle \mathrm{SOMO}\;\left|\mathrm{SOMO}\right.\right\rangle$ and energy gap Δ*ϵ* (in eV), and e–f) overlaps S between the highest occupied orbitals $\left\langle \mathrm{HOMO}-1\;\left|\mathrm{SOMO}\right.\right\rangle$ and $\left\langle \mathrm{HOMO}-1\;\left|\mathrm{HOMO}-1\right.\right\rangle$ in the A_1_ orbital interaction scheme (Figure S4), as a function of the bond distance of H_3_C−YH_n_ (left) and H_3_Si−YH_n_ (right) with YH_n_=F and Cl (equilibrium geometry indicated with a dot), computed at BLYP‐D3(BJ)/TZ2P.

**Figure 4:**
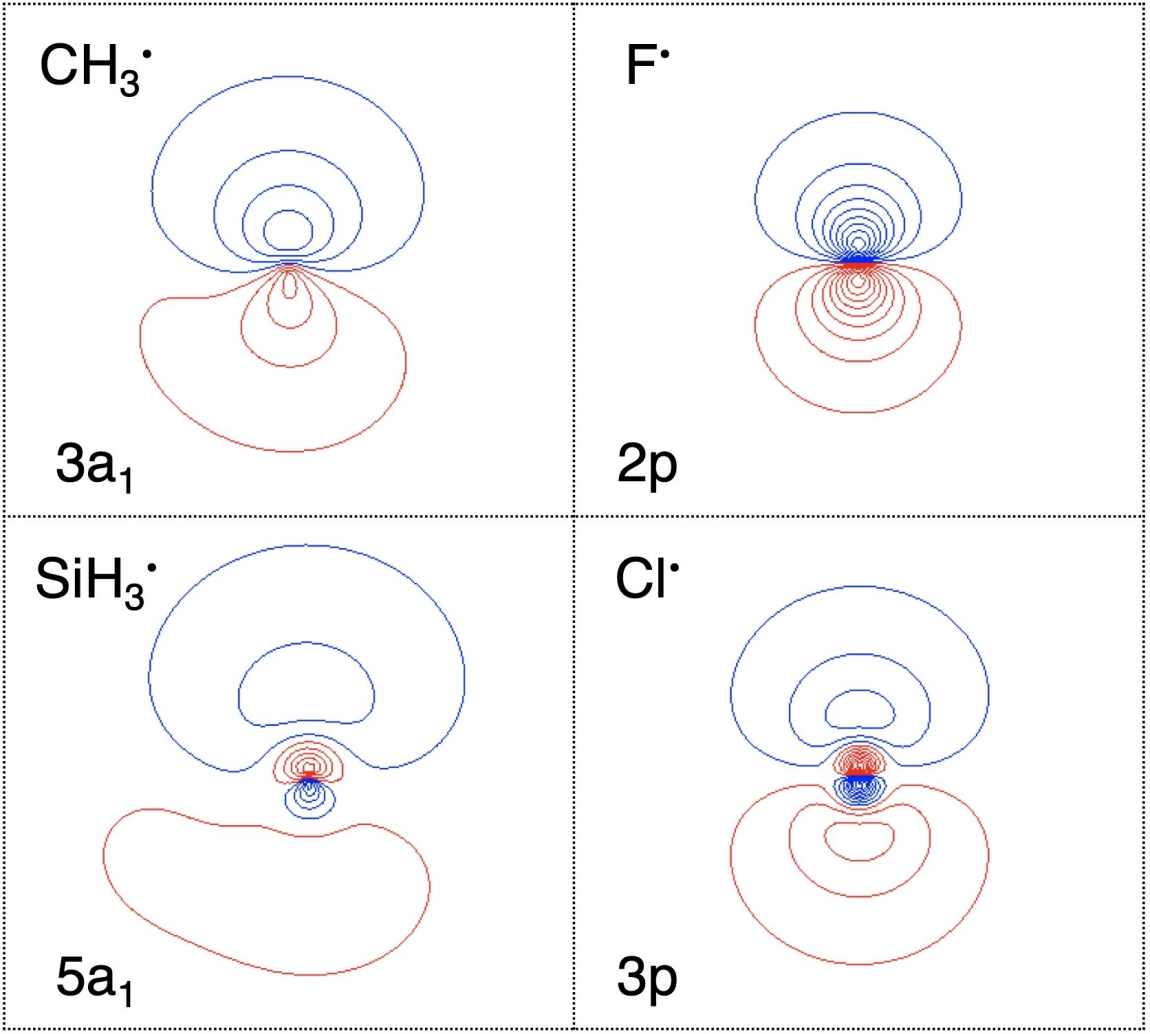
Contour plots of CH_3_^.^, F^.^, SiH_3_^.^, and Cl^.^ SOMOs (10 contour lines between 0.05, 1.0; scan values are evenly spaced; color represents phase), computed at BLYP‐D3(BJ)/TZ2P.

But why does Δ*E*_int_, and thus the BDE, become weaker from C−F to C−Cl? The reason appears to be the increase in effective atom size of the halogen and, thus, the increase in Pauli repulsion Δ*E*_Pauli_ (Figure 3a) if one goes from F to Cl. The latter has spatially more extended occupied valence AOs which leads to an increase in the occupied−occupied overlap S (Figure 3e). Also, the heavier halogen has more subvalence shells which further raise the number of Pauli repulsive occupied−occupied orbital interactions. For example, at a consistent bond distance of 1.400 Å (Table 2), Δ*E*_Pauli_ increases from 264.9 to 567.6 kcal mol^−1^ along C−F to C−Cl. This does not only make the carbon−halogen bond weaker but of course also pushes it to a longer equilibrium distance, from 1.413 Å for C−F to 1.820 Å for C−Cl (Figure 3a). Eventually, at this longer equilibrium distance, all energy terms are weaker. Interestingly, this leads to Δ*E*_oi_ becoming less stabilizing at the respective equilibrium bond distances if we go from C−F to C−Cl (−272.5 to −167.4 kcal mol^−1^, Table 2). Note that this trend Δ*E*_oi_ at the equilibrium bond distances does *not* originate from the decrease in SOMO−SOMO gap Δ*ϵ*, and occurs despite an increase in bond overlap. It is a side effect of the increased Δ*E*_Pauli_, and the resulting longer C−X bond, for the larger halogen. This trend, as well as the underlying mechanism, continues along the whole series of carbon‐halogen bonds, with BDEs decreasing from 111.3 to 80.9 to 71.2 to 61.0 kcal mol^−1^ along C−F, C−Cl, C−Br, and C−I (see Figures S5 and S6).[^27^, ^28^]

The same mechanism is found for the silicon−halogen bonds. From Si−F to Si−Cl, the Δ*E*_int_ becomes less stable from −151.1 to −105.6 kcal mol^−1^, and the bond lengthens from 1.625 to 2.082 Å, respectively. Down the halogens, the Δ*E*_oi_ in the Si−X bond (Figure 3b) becomes more stable due to the increase SOMO−SOMO overlap S, and despite the decrease in energy gap Δ*ϵ* (Figure 3d). Again, the increase in Δ*E*_Pauli_ from Si−F to Si−Cl (Figure 3b) is what determines the trend in bond strength (and length) because of the increase in occupied−occupied overlap S (Figure 3f) as well as the larger number of subvalence shells in the case of the heavier halogen. Likewise, the series Cl−F to Cl−Cl and H−F to H−Cl (which augments work in Ref. [29]) reveal the same trends and mechanism (Figures S2, S3, and S7).

The popular electronegativity model, however, does not break down in all cases. In particular, the trend in X−Y bond strength as one of the atoms runs along a period (instead of down a group) does indeed depend in a causal way on the trend in electronegativity (Figure 2c), but also on Pauli repulsive closed‐shell interactions. For example, from C−C to C−F, the bond energy Δ*E* strengthens from −92.1 to −115.3 kcal mol^−1^ (see Table 2) because of a corresponding trend in Δ*E*_int_ (strengthening from −110.4 to −121.6 kcal mol^−1^), modulated by the strain energy Δ*E*_strain_ associated with pyramidalizing one or two methyl groups.[^24^, ^26^] The strengthening in Δ*E*_int_ from C−C to C−F is determined by both Δ*E*_Pauli_ and Δ*E*_oi_ and somewhat counteracted by Δ*V*_elstat_ (Figure 5a). The Δ*E*_Pauli_ becomes less repulsive along this series due to the smaller occupied valence atomic orbitals for fluorine, which decreases the occupied−occupied overlap S (Figure 5e). The Δ*E*_oi_ becomes more stabilizing (Figure 5a), and is, especially at the C−F equilibrium, essential to overcome the destabilization in Δ*V*_elstat_. The stabilization in Δ*E*_oi_ is caused by the larger, more favorable SOMO−SOMO gap Δ*ϵ* for the C−F bond (Figures 2c and 5c), and despite the reduction in bond overlap that emerges from the aggravating mismatch in spatial extension between the SOMOs from C−C to C−F (Figure 4). Likewise, we find that the same trends and underlying bonding mechanism are active for the analogous series along a period, for example, along Si−C to Si−F bonds (Figure 5b, d, f), as well as for Si−Si to Si−Cl (Figures S2 and S3).

**Figure 5:**
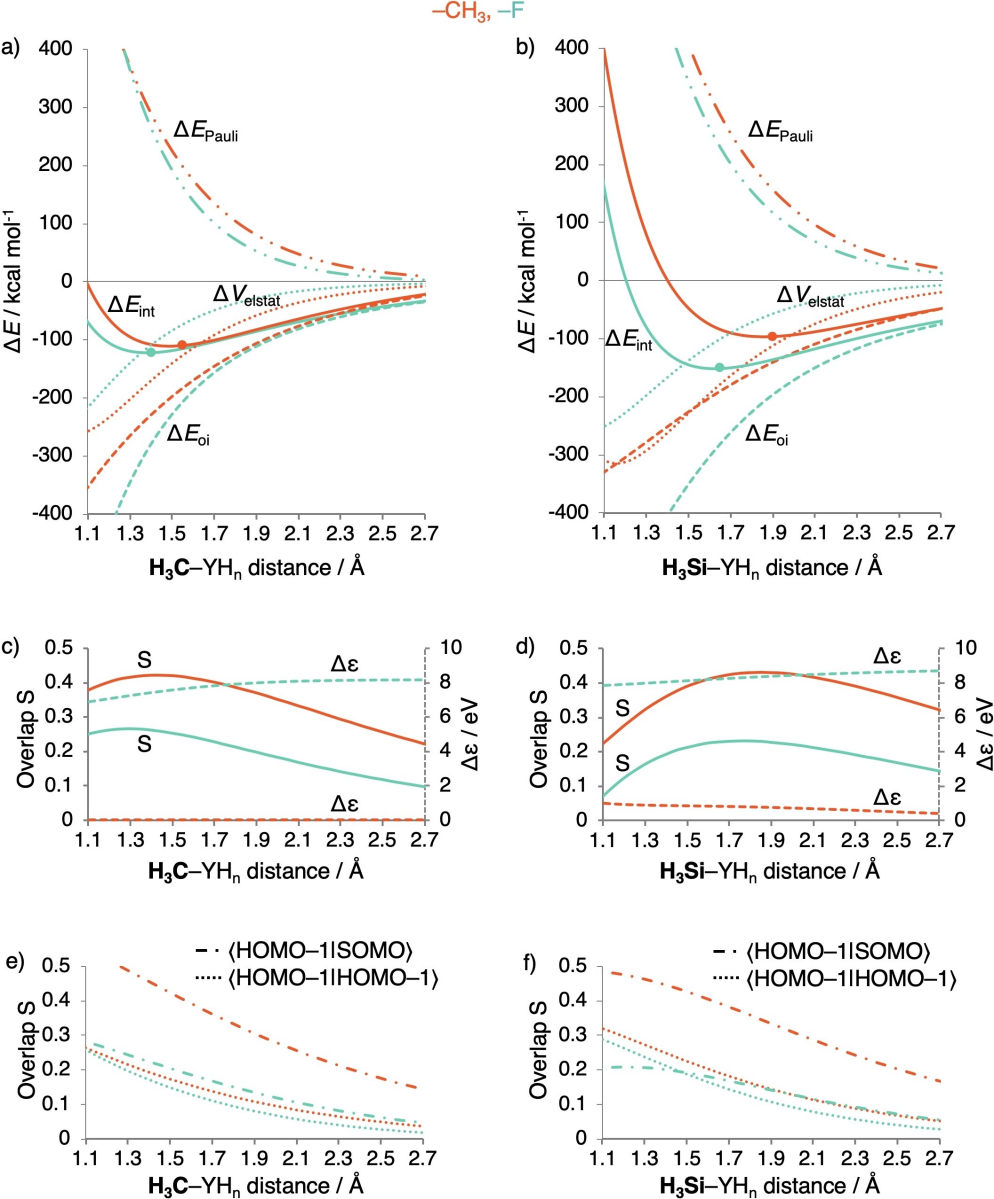
a–b) Energy decomposition analysis, c–d) SOMO−SOMO overlap S $\left\langle \mathrm{SOMO}\;\left|\mathrm{SOMO}\right.\right\rangle$ and energy gap Δ*ϵ* (in eV), and e–f) overlaps S between the highest occupied orbitals $\left\langle \mathrm{HOMO}-1\;\left|\mathrm{SOMO}\right.\right\rangle$ and $\left\langle \mathrm{HOMO}-1\;\left|\mathrm{HOMO}-1\right.\right\rangle$ in the A_1_ orbital interaction scheme (Figure S4), as a function of the bond distance of H_3_C−YH_n_ (left) and H_3_Si−YH_n_ (right) with YH_n_=CH_3_ and F (equilibrium geometry indicated with a dot), computed at BLYP‐D3(BJ)/TZ2P.

We already mentioned that the simple trend of stronger BDE for larger Δχ is in some cases disturbed, notably from C−C to C−N, along which Δ*E* weakens, instead of strengthens, from −92.1 to −87.3 kcal mol^−1^ (see Figure 1 for the irregularity). This anomaly is caused by the pyramidalization of either two or one methyl group(s) (C−C versus C−N).^[24]^ The C−C bond experiences a stabilizing effect, since the cost of Δ*E*_strain_ upon pyramidalizing two methyl groups goes with an even larger relief of steric (Pauli) repulsion, as the C−H bonds of one methyl fragment bend away from the other fragment, and vice versa, causing the C−C bond to be stronger than the C−N bond. However, pyramidalization is a special case for methyl groups, and does not, or to a lesser extent, occur for other fragments that are already pyramidal, such as SiH_3_,^[26]^ or that have lone‐pair orbitals at the central atom that do not contain substituents to bend away, for instance for NH_2_, OH, or F.^[24]^

In conclusion, we have shown that the correlation between the electron‐pair bond strength and the electronegativity difference across the bond is not always causal. One of the striking exceptions is the series of carbon−halogen bonds which, ironically, is a popular, but erroneous as we show, example in textbooks for illustrating the aforementioned electronegativity model. Instead, we show that the carbon−halogen bond weakens from C−F to C−I because of an increasing steric (Pauli) repulsion with the effectively larger atom size and electron‐richer heavier halogen atoms. This bond weakening from C−F to C−I occurs *despite* an orbital interaction which, at any given bond distance, becomes stronger, not weaker, because of an increasing bond overlap between the relatively diffuse methyl SOMO and the increasingly diffuse halogen n*p* SOMO. Interestingly, it is the buildup of Pauli repulsion that, for heavier halogens, pushes the C−X bond to a longer equilibrium bond distance at which the orbital interaction becomes weaker, thus, establishing the non‐causal correlation with the decreasing electronegativity difference. Finally, our work also shows that, for a full understanding of the causalities in a bonding mechanism, it is crucial to carry out the bonding analyses as a function of the bond formation process.

## Conflict of interest

The authors declare no conflict of interest.

## Supporting information

As a service to our authors and readers, this journal provides supporting information supplied by the authors. Such materials are peer reviewed and may be re‐organized for online delivery, but are not copy‐edited or typeset. Technical support issues arising from supporting information (other than missing files) should be addressed to the authors.

Supporting Information
