# Supplementary material for: The Chemical Bond: When Atom Size Instead of Electronegativity Difference Determines Trend in Bond Strength
Source: Chemistry. 2021 Oct 19;27(63):15616–22. doi: 10.1002/chem.202103544 (PMC9298008; doi:10.1002/chem.202103544)
Supplement: Supplementary file 1 — Supporting Information [file CHEM-27-15616-s001.pdf]

Chemistry–A European Journal

Supporting Information

The Chemical Bond: When Atom Size Instead of Electronegativity Difference Determines Trend in Bond Strength

Eva Blokker, Xiaobo Sun, Jordi Poater, J. Martijn van der Schuur, Trevor A. Hamlin, and F. Matthias Bickelhaupt*

Contents

Computational Methods

Table S1. Bond dissociation energies, enthalpies and Gibbs free energies of all $H_nX–YH_n$ systems in this study. Thermodynamic data at 298.15 K and 1 atm.

Table S2. Activation strain analysis terms (in kcal mol^{-1}) and bond lengths (in Å) of all $H_nX–YH_n$ systems in this study.

Figure S1. Activation strain analysis as a function of the bond distance of a) $H_3C–YH_n$, b) $F–YH_n$, c) $SiH_3–YH_n$, and d) $Cl–YH_n$ (YH_n = CH_3, F, SiH_3, and Cl).

Figure S2. Energy decomposition analysis as a function of the distance of a) $H_3C–YH_n$, b) $F–YH_n$, c) $H_3Si–YH_n$, and d) $Cl–YH_n$ (YH_n = CH_3, F, SiH_3, and Cl).

Figure S3. SOMO–SOMO overlap S ⟨SOMO|SOMO⟩ and gap $\Delta\varepsilon$ as a function of the distance of a) $H_3C–YH_n$, b) $F–YH_n$, c) $H_3Si–YH_n$, and d) $Cl–YH_n$ (YH_n = CH_3, F, SiH_3, and Cl).

Table S3. Decomposition of the difference in electrostatic interaction $\Delta\Delta V_{elstat}$ (in kcal mol^{-1}) at equal distance (1.650 Å) when going from $H_nX–CH_3$ to $H_nX–F$ (upper) and from $H_nX–F$ to $H_nX–Cl$ (bottom) (H_nX = H_3C and H_3Si).

Figure S4. Quantitative A_1 orbital interaction scheme of a) $H_3C–CH_3$, b) $H_3C–F$, c) $H_3C–Cl$, d) $H_3C–SiH_3$, e) $H_3Si–F$, and f) $H_3Si–Cl$, at the equilibrium geometry. For $F^•$, the 2s orbital (HOMO–1) at –30.5 eV is not shown.

Figure S5. a) Activation strain analysis, b) energy decomposition analysis, and c) SOMO–SOMO overlap S and gap $\Delta\varepsilon$ as a function of the $H_3C–Y$ distance (Y = F, Cl, Br, and I).

Figure S6. a) Activation strain analysis, b) energy decomposition analysis, and c) SOMO–SOMO overlap S and gap $\Delta\varepsilon$ as a function of the $H_3C–Y$ distance (Y = F, Cl, Br, and I), computed at relativistic ZORA-BLYP-D3(BJ)/TZ2P.

Figure S7. a) Energy decomposition analysis and b) SOMO–SOMO overlap S and gap $\Delta\varepsilon$ as a function of the H–Y distance (Y = F and Cl).

Table S4. Cartesian coordinates (in Å), ADF total bonding energies [TBE] (in kcal mol^{-1}), and number of imaginary frequencies NIMAG of the equilibrium geometries of all $H_nX–YH_n$ complexes in this study.

Table S5. Cartesian coordinates (in Å), ADF total bonding energies [TBE] (in kcal mol^{-1}), and number of imaginary frequencies NIMAG of the equilibrium geometries of all radical species in this study.

Computational Methods

Computational details

All calculations were performed with the Amsterdam Density Functional (ADF) program.[1,2] Molecular orbitals (MOs) were expanded using a large uncontracted set of Slater-type orbitals (STO): TZ2P.[3] The TZ2P basis set is of triple-ζ quality, augmented by two sets of polarization functions. All electrons were treated variationally. The Generalized Gradient Approximation (GGA) functional BLYP[4] with Grimme-D3 dispersion-corrections and finite damping introduced by Becke and Johnson (BJ),[5] BLYP-D3(BJ), was used for calculating the geometries and energies, for Br and I including relativistic effects computed using scalar ZORA.[6] Note that differences between carbon–halogen bond strengths computed without and with ZORA relativistic effects are always smaller than 1 kcal mol^{-1}, as shown in the work of de Jong et al.[7] Also, compare the nearly identical results of nonrelativistic and relativistic carbon–halogen bonding analyses in Figures S5 and S6. No geometry restrictions were used unless otherwise stated. The radical fragments were treated with a spin-unrestricted formalism and the open-shell PyFrag2019 program was used to analyze the bond dissociation as a function of the $H_nX–YH_n$ distance.[8]

Thermochemistry

Enthalpies at 298.15 K and 1 atmosphere (ΔH_{298}) were calculated from electronic bond energies (ΔE) and vibrational frequencies using standard thermochemistry relations for an ideal gas, according to Equation (2):[9]

$$\Delta H_{298} = \Delta E + \Delta E_{\text{trans},298} + \Delta E_{\text{rot},298} + \Delta E_{\text{vib},0} + \Delta(\Delta E_{\text{vib},0})_{298} + \Delta(pV) \tag{2}$$

Here, $\Delta E_{\text{trans},298}$, $\Delta E_{\text{rot},298}$ and $\Delta E_{\text{vib},0}$ are the differences between the reactant (i.e., $H_nX–YH_n$, the complex) and products (i.e., $XH_n^{\bullet}$ and $YH_n^{\bullet}$, the radical main-group-element hydrides) in translational, rotational and zero-point vibrational energy, respectively. $\Delta(\Delta E_{\text{vib},0})_{298}$ is the change in the vibrational energy difference as one goes from 0 to 298.15 K. The vibrational energy corrections are based on our frequency calculations. The molar work term $\Delta(pV)$ is $(\Delta n)RT$; $\Delta n = +1$ for one reactant $H_nX–YH_n$ dissociating into the two radical products $XH_n^{\bullet}$ and $YH_n^{\bullet}$. Thermal corrections for the electronic energy are neglected.

Activation strain and energy decomposition analysis

For the activation strain analysis (ASA), the overall X–Y bond energy ΔE [which also features in Eq. (2)] between the radicals $XH_n^{\bullet}$ and $YH_n^{\bullet}$ in $H_nX–YH_n$ is made up of two major components:[10]

$$\Delta E = \Delta E_{\text{strain}} + \Delta E_{\text{int}} \tag{3}$$

Here, the strain energy ΔE_{strain} is the amount of energy required to deform the radical fragments from their equilibrium structure to the geometry that they acquire in the overall

complex $H_nX–YH_n$. The fluorine and chlorine radicals have zero strain energy, since they consist solely of one atom and therefore cannot undergo geometrical deformations. The interaction energy ΔE_{int} corresponds to the actual energy change when the geometrically deformed fragments are combined to form the overall complex.

We further analyze the interaction ΔE_{int} in the framework of the canonical Kohn-Sham molecular orbital (MO) model, by dissecting it through our canonical energy decomposition analyses (canonical EDA) into electrostatic attraction, Pauli repulsion, (attractive) orbital interactions, the dispersion energy and the spin polarization:[1, 10]

$$\Delta E_{int} = \Delta V_{elstat} + \Delta E_{Pauli} + \Delta E_{oi} + \Delta E_{disp} + \Delta E_{spinpol} \quad (4)$$

The term ΔV_{elstat} corresponds to the classical electrostatic interaction between the unperturbed charge distributions of the radical fragments $XH_n^{\bullet}$ and $YH_n^{\bullet}$ in the geometry they possess in $H_nX–YH_n$. This term is usually attractive. The Pauli repulsion ΔE_{Pauli} between these fragments comprises the destabilizing interactions between occupied orbitals (or, more precisely, spinorbitals of equal spin) on either fragment and is responsible for the steric repulsion. The orbital interaction ΔE_{oi} between these fragments in any MO model, and therefore also in Kohn-Sham theory, accounts for electron-pair bonding (the SOMO–SOMO interaction), charge transfer (empty/occupied orbital mixing between different fragments), and polarization (empty/occupied orbital mixing on one fragment due to the presence of another fragment). The dispersion energy ΔE_{disp} is added as a correction.[5] Finally, the term $\Delta E_{spinpol}$ refers to the spin polarization of the spin-α and spin-β electrons of the deformed unrestricted fragments and is with respect to ΔE_{int} destabilizing (i.e., the deformed unrestricted fragments *without* spin polarization lie consistently 2-3 kcal mol^{-1} higher in energy and therefore have a too stabilizing ΔE_{int}).[11]

References.

[1] a) G. te Velde, F. M. Bickelhaupt, E. J. Baerends, C. Fonseca Guerra, S. J. A. van Gisbergen, J. G. Snijders, T. Ziegler, *J. Comput. Chem.* **2001**, *22*, 931; b) C. Fonseca Guerra, J. G. Snijders, G. te Velde, E. J. Baerends, *Theor. Chem. Acc.* **1998**, *99*, 391; c) ADF2017, SCM, Theoretical Chemistry, Vrije Universiteit Amsterdam (The Netherlands), http://www.scm.com.

[2] G. te Velde, F. M. Bickelhaupt, E. J. Baerends, C. Fonseca Guerra, S. J. A. van Gisbergen, J. G. Snijders, T. Ziegler, *J. Comput. Chem.* **2001**, *22*, 931.

[3] E. van Lenthe, E. J. Baerends, *J. Comput. Chem.* **2003**, *24*, 1142.

[4] a) A. D. Becke, *Phys. Rev. A.* **1988**, *38*, 3098; b) C. T. Lee, W. T. Yang, R. G. Parr, *Phys. Rev. B.* **1988**, *37*, 785.

[5] a) S. Grimme, J. Antony, S. Ehrlich, H, Krieg, *J. Chem. Phys.* **2010**, *132*, 154104; b) S. Grimme, S. Ehrlich, L. Goerigk, *J. Comput. Chem.* **2011**, *32*, 1456.

[6] a) E. van Lenthe, E. J. Baerends, J. G. Snijders, *J. Chem. Phys.* **1993**, *99*, 4597; b) E. van Lenthe, E. J. Baerends, J. G. Snijders, *J. Chem. Phys.* **1994**, *101*, 9783.

[7] G. T. de Jong, F. M. Bickelhaupt, *J. Chem. Theory Comput.* **2007**, *3*, 514.

[8] a) W. -J. van Zeist, C. Fonseca Guerra, F. M. Bickelhaupt, *J. Comp. Chem.* **2008**, *29*,

312; b) X. Sun, T. M. Soini, J. Poater, T. A. Hamlin, F. M. Bickelhaupt, *J. Comp. Chem.* **2019**, *40*, 2227.

[9] a) P. W. Atkins, J. de Paula, *Physical Chemistry*, 9th ed., W. H. Freeman, New York **2010**; b) F. Jensen, *Introduction to Computational Chemistry*, Wiley, New York **2007**.

[10] a) F. M. Bickelhaupt, E. J. Baerends, in *Reviews in Computational Chemistry*, (Eds.: K. B. Lipkowitz, D. B. Boyd), Wiley–VCH, Hoboken **2000**, pp 1–86; b) T. A. Hamlin, P. Vermeeren, C. Fonseca Guerra, F. M. Bickelhaupt, in *Complementary Bonding Analysis* (Ed: S. Grabowsky), De Gruyter, Berlin **2021**, pp 199–212; c) T. Ziegler, A. Rauk, *Theoret. Chim. Acta.* **1977**, *46*, 1; d) T. Ziegler, A. Rauk, *Inorg. Chem.* **1979**, *18*, 1755.

[11] F. M. Bickelhaupt, M. Solà, C. Fonseca Guerra, *Faraday Discuss.* **2007**, *135*, 451.

Table S1. Bond dissociation energies, enthalpies and Gibbs free energies of all $H_nX{-}YH_n$ systems in this study, computed at BLYP-D3(BJ)/TZ2P. Thermodynamic data at 298.15 K and 1 atm.

	$YH_n^{\bullet}$											
	$CH_3^{\bullet}$			$NH_2^{\bullet}$			$OH^{\bullet}$			$F^{\bullet}$		
$H_nX^{\bullet}$	ΔE	ΔH	ΔG	ΔE	ΔH	ΔG	ΔE	ΔH	ΔG	ΔE	ΔH	ΔG
$H_3C^{\bullet}$	92.1	85.2	74.6	87.3	80.3	70.5	95.1	89.2	80.5	115.3	111.3	103.4
$H_2N^{\bullet}$	87.3	80.3	70.5	67.2	60.7	50.0	65.4	59.8	50.8	77.7	74.2	66.8
$HO^{\bullet}$	95.1	89.2	80.5	65.4	59.8	50.8	57.0	52.9	44.6	57.9	55.9	49.4
$F^{\bullet}$	115.3	111.3	103.4	77.7	74.2	66.8	57.9	55.9	49.4	48.5	48.0	41.7
$H_3Si^{\bullet}$	87.5	83.2	72.9	101.4	96.7	87.1	122.0	118.1	109.2	151.1	148.6	140.2
$H_2P^{\bullet}$	72.1	67.3	57.5	72.6	68.3	57.8	90.7	86.5	77.5	114.2	112.0	104.3
$HS^{\bullet}$	75.0	70.7	62.1	68.3	63.9	54.8	72.7	69.5	61.3	87.5	86.2	79.6
$Cl^{\bullet}$	84.2	80.9	73.3	63.9	60.8	53.7	59.5	57.8	51.5	67.0	66.8	61.1

	$YH_n^{\bullet}$											
	$SiH_3^{\bullet}$			$PH_2^{\bullet}$			$SH^{\bullet}$			$Cl^{\bullet}$		
$H_nX^{\bullet}$	ΔE	ΔH	ΔG	ΔE	ΔH	ΔG	ΔE	ΔH	ΔG	ΔE	ΔH	ΔG
$H_3C^{\bullet}$	87.5	83.2	72.9	72.1	67.3	57.5	75.0	70.7	62.1	84.2	80.9	73.3
$H_2N^{\bullet}$	101.4	96.7	87.1	72.6	68.3	57.8	68.3	63.9	54.8	63.9	60.8	53.7
$HO^{\bullet}$	122.0	118.1	109.2	90.7	86.5	77.5	72.7	69.5	61.3	59.5	57.8	51.5
$F^{\bullet}$	151.1	148.6	140.2	114.2	112.0	104.3	87.5	86.2	79.6	67.0	66.8	61.1
$H_3Si^{\bullet}$	74.3	71.4	60.9	69.6	66.4	56.6	85.1	82.3	73.4	105.5	103.6	95.6
$H_2P^{\bullet}$	69.6	66.4	56.6	57.9	54.4	44.5	66.3	63.3	54.4	77.7	75.9	68.5
$HS^{\bullet}$	85.1	82.3	73.4	66.3	63.3	54.4	63.9	61.7	53.1	64.7	63.8	57.6
$Cl^{\bullet}$	105.5	103.6	95.6	77.7	75.9	68.5	64.7	63.8	57.6	59.2	59.2	53.3

Table S2. Activation strain analysis terms (in kcal mol^{-1}) and bond lengths (in Å) of all H_nX–YH_n systems in this study, computed at BLYP-D3(BJ)/TZ2P.

	$YH_n^{\bullet}$							
	$CH_3^{\bullet}$				$NH_2^{\bullet}$			
$H_nX^{\bullet}$	ΔE	ΔE_{strain}	ΔE_{int}	d	ΔE	ΔE_{strain}	ΔE_{int}	d
$H_3C^{\bullet}$	–92.1	18.4	–110.4	1.538	–87.3	9.4	–96.7	1.478
$H_2N^{\bullet}$	–87.3	9.4	–96.7	1.478	–67.2	0.2	–67.4	1.511
$HO^{\bullet}$	–95.1	8.1	–103.2	1.442	–65.4	0.4	–65.7	1.466
$F^{\bullet}$	–115.3	6.3	–121.6	1.413	–77.7	0.1	–77.8	1.470
$H_3Si^{\bullet}$	–87.5	9.1	–96.6	1.888	–101.4	1.4	–102.8	1.737
$H_2P^{\bullet}$	–72.1	8.0	–80.1	1.882	–72.6	0.2	–72.8	1.787
$HS^{\bullet}$	–75.0	7.1	–82.2	1.846	–68.3	1.0	–69.2	1.729
$Cl^{\bullet}$	–84.2	5.9	–90.0	1.820	–63.9	0.2	–64.0	1.800

	$YH_n^{\bullet}$							
	$OH^{\bullet}$				$F^{\bullet}$			
$H_nX^{\bullet}$	ΔE	ΔE_{strain}	ΔE_{int}	d	ΔE	ΔE_{strain}	ΔE_{int}	d
$H_3C^{\bullet}$	–95.1	8.1	–103.2	1.442	–115.3	6.3	–121.6	1.413
$H_2N^{\bullet}$	–65.4	0.4	–65.7	1.466	–77.7	0.1	–77.8	1.470
$HO^{\bullet}$	–57.0	0.1	–57.1	1.494	–57.9	0.0	–58.0	1.473
$F^{\bullet}$	–57.9	0.0	–58.0	1.473	–48.5	0.0	–48.5	1.439
$H_3Si^{\bullet}$	–122.0	0.4	–122.3	1.669	–151.1	0.0	–151.1	1.625
$H_2P^{\bullet}$	–90.7	0.1	–90.8	1.691	–114.2	0.0	–114.2	1.643
$HS^{\bullet}$	–72.7	0.1	–72.8	1.706	–87.5	0.0	–87.5	1.664
$Cl^{\bullet}$	–59.5	0.0	–59.5	1.744	–67.0	0.0	–67.0	1.683

	$YH_n^{\bullet}$							
	$SiH_3^{\bullet}$				$PH_2^{\bullet}$			
$H_nX^{\bullet}$	ΔE	ΔE_{strain}	ΔE_{int}	d	ΔE	ΔE_{strain}	ΔE_{int}	d
$H_3C^{\bullet}$	–87.5	9.1	–96.6	1.888	–72.1	8.0	–80.1	1.882
$H_2N^{\bullet}$	–101.4	1.4	–102.8	1.737	–72.6	0.2	–72.8	1.787
$HO^{\bullet}$	–122.0	0.4	–122.3	1.669	–90.7	0.1	–90.8	1.691
$F^{\bullet}$	–151.1	0.0	–151.1	1.625	–114.2	0.0	–114.2	1.643
$H_3Si^{\bullet}$	–74.3	0.6	–74.8	2.356	–69.6	0.3	–69.9	2.288
$H_2P^{\bullet}$	–69.6	0.3	–69.9	2.288	–57.9	0.0	–57.9	2.281
$HS^{\bullet}$	–85.1	0.1	–85.2	2.168	–66.3	0.0	–66.3	2.169
$Cl^{\bullet}$	–105.5	0.1	–105.6	2.082	–77.7	0.0	–77.7	2.112

	$YH_n^{\bullet}$							
	$SH^{\bullet}$				$Cl^{\bullet}$			
$H_nX^{\bullet}$	ΔE	ΔE_{strain}	ΔE_{int}	d	ΔE	ΔE_{strain}	ΔE_{int}	d
$H_3C^{\bullet}$	–75.0	7.1	–82.2	1.846	–84.2	5.9	–90.0	1.820
$H_2N^{\bullet}$	–68.3	1.0	–69.2	1.729	–63.9	0.2	–64.0	1.800
$HO^{\bullet}$	–72.7	0.1	–72.8	1.706	–59.5	0.0	–59.5	1.744
$F^{\bullet}$	–87.5	0.0	–87.5	1.664	–67.0	0.0	–67.0	1.683
$H_3Si^{\bullet}$	–85.1	0.1	–85.2	2.168	–105.5	0.1	–105.6	2.082
$H_2P^{\bullet}$	–66.3	0.0	–66.3	2.169	–77.7	0.0	–77.7	2.112
$HS^{\bullet}$	–63.9	0.0	–63.9	2.102	–64.7	0.0	–64.7	2.083
$Cl^{\bullet}$	–64.7	0.0	–64.7	2.083	–59.2	0.0	–59.2	2.048

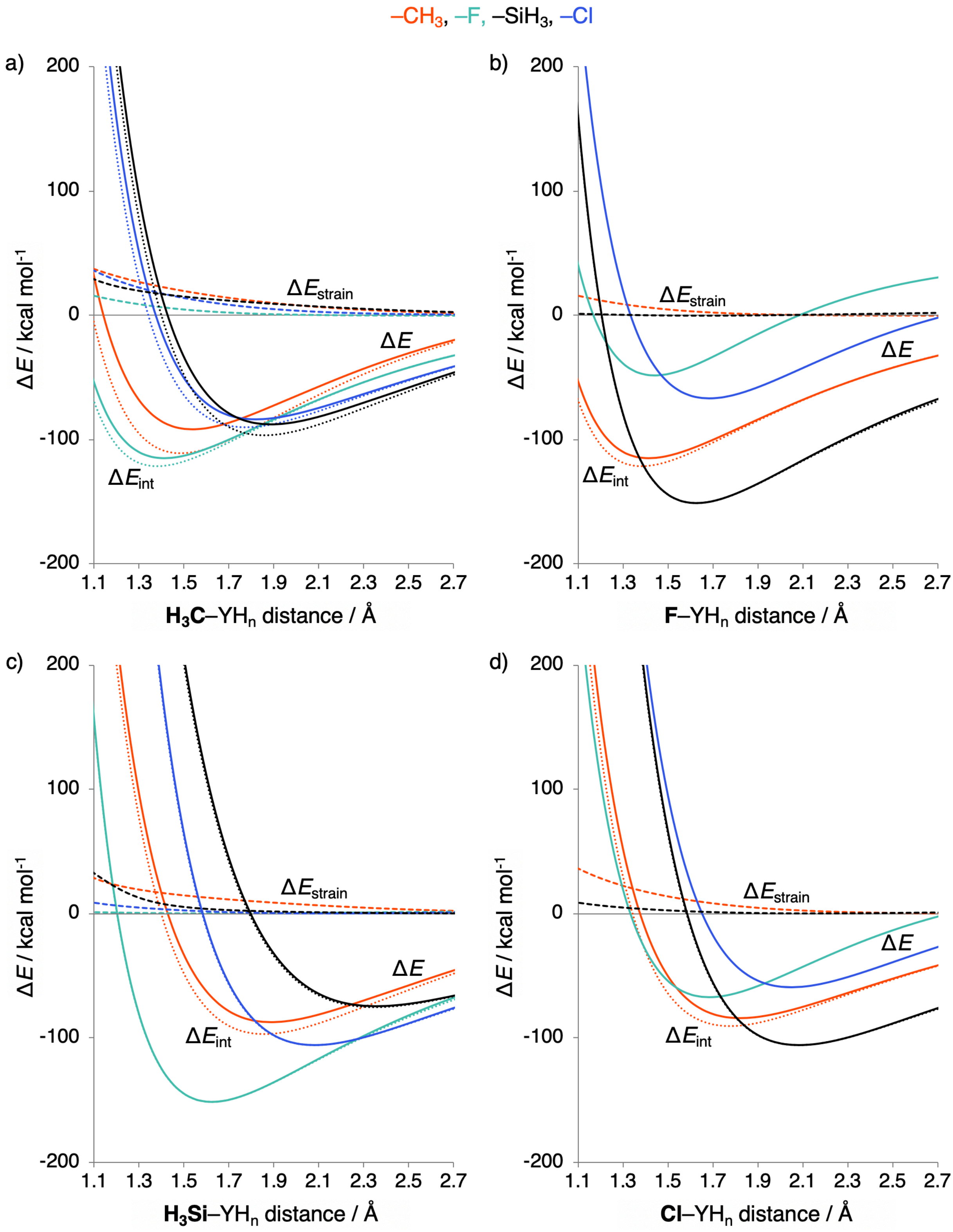

Figure S1. Activation strain analysis as a function of the bond distance of a) $H_3C–YH_n$, b) F–YH_n, c) $H_3Si–YH_n$, and d) Cl–YH_n (YH_n = CH_3, F, SiH_3, and Cl), computed at BLYP-D3(BJ)/TZ2P.

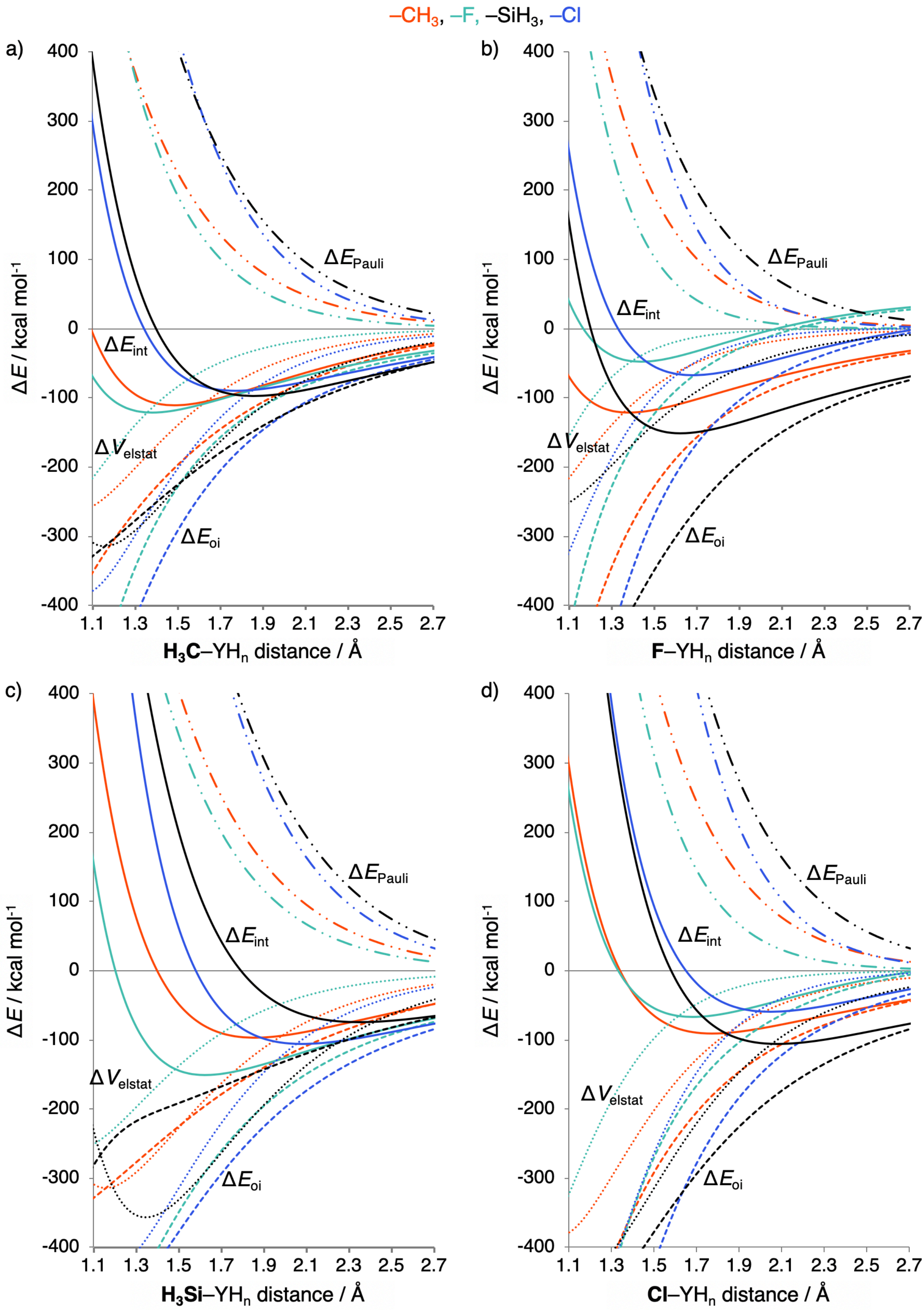

Figure S2. Energy decomposition analysis as a function of the distance of a) H_3C–YH_n, b) F–YH_n, c) H_3Si–YH_n, and d) Cl–YH_n (YH_n = CH_3, F, SiH_3, and Cl), computed at BLYP-D3(BJ)/TZ2P.

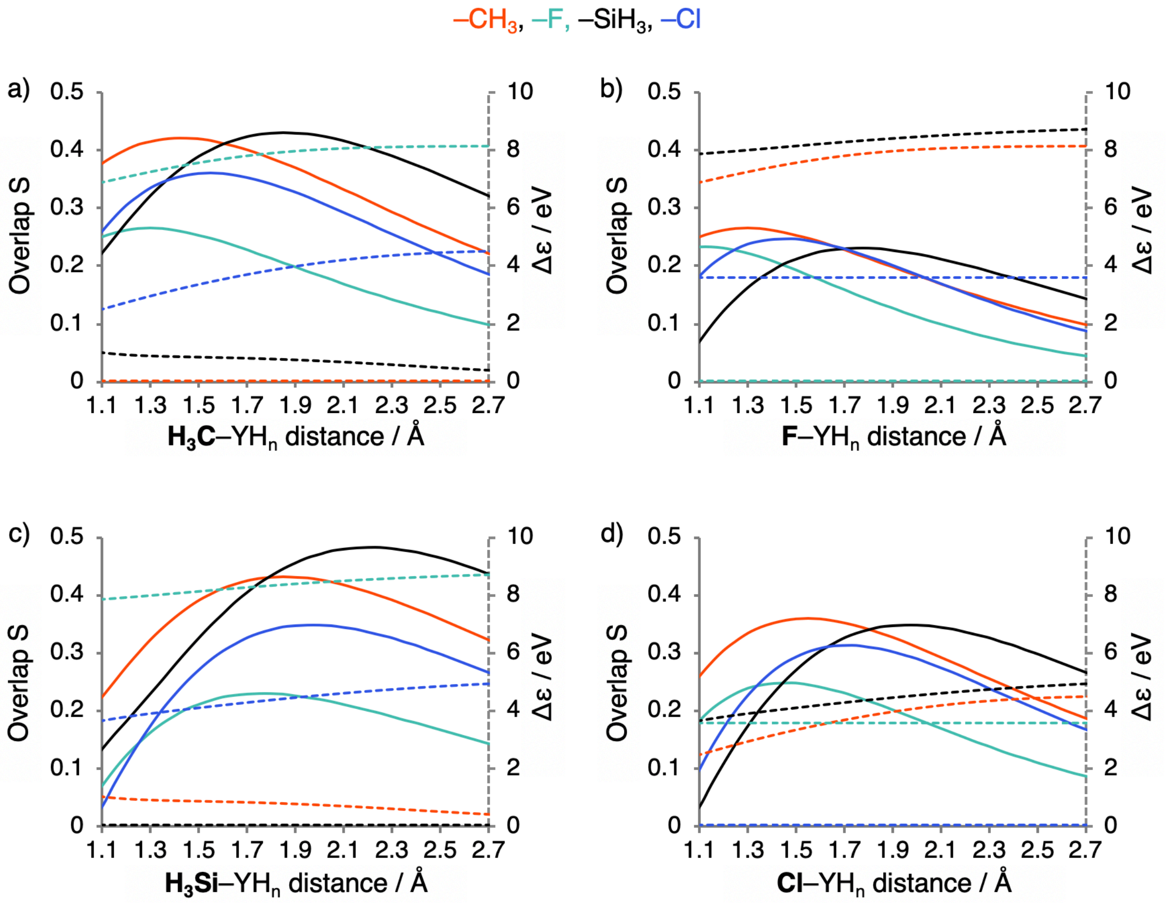

Figure S3. SOMO–SOMO overlap S ⟨SOMO|SOMO⟩ and gap Δε as a function of the distance of a) H_3C–YH_n, b) F–YH_n, c) H_3Si–YH_n, and d) Cl–YH_n (YH_n = CH_3, F, SiH_3, and Cl), computed at BLYP-D3(BJ)/TZ2P.

Table S3. Decomposition of the difference in electrostatic interaction $\Delta\Delta V_{\mathrm{elstat}}$ (in kcal mol^{-1}) at equal distance (1.650 Å) when going from H_nX–CH_3 to H_nX–F (upper) and from H_nX–F to H_nX–Cl (bottom) (H_nX = H_3C and H_3Si), computed at BLYP-D3(BJ)/TZ2P.

	$\Delta\Delta V_{\mathrm{elstat}}$	Δe–e	ΔN–N	Δe–N	ΔN–e
H_3C–CH_3 to H_3C–F	+46.0	+1246.4	+1389.6	–1397.1	–1187.9
H_3Si–CH_3 to H_3Si–F	+77.7	+2355.3	+2705.7	–2648.2	–2326.9

	$\Delta\Delta V_{\mathrm{elstat}}$	Δe–e	ΔN–N	Δe–N	ΔN–e
H_3C–F to H_3C–Cl	–88.3	+12763.3	+12936.6	–13001.7	–12791.1
H_3Si–F to H_3Si–Cl	–141.9	+24645.7	+25451.0	–25111.2	–25132.6

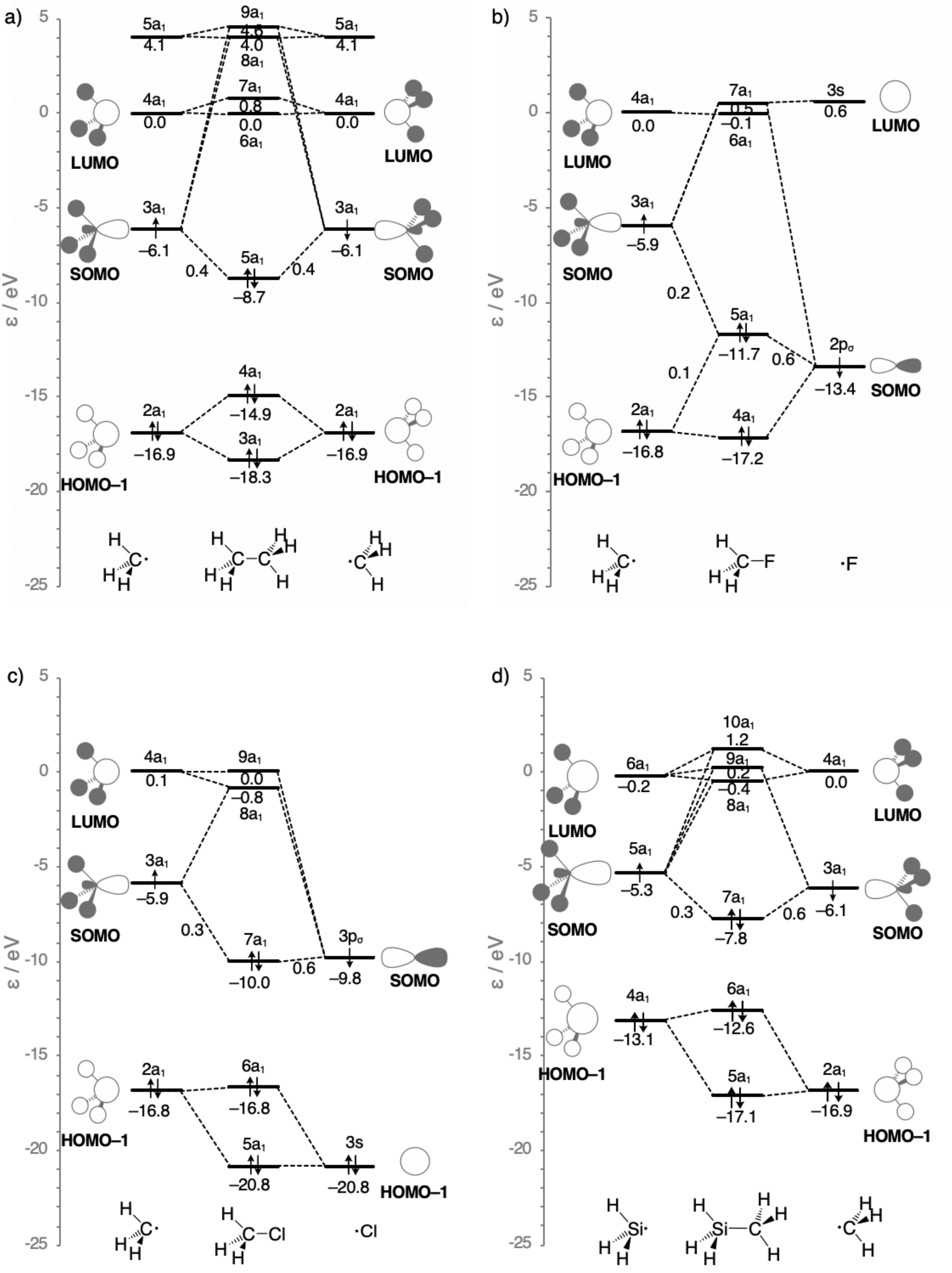

Figure S4. Quantitative A_1 orbital interaction scheme of a) $H_3C–CH_3$, b) $H_3C–F$, c) $H_3C–Cl$, d) $H_3C–SiH_3$, e) $H_3Si–F$, and f) $H_3Si–Cl$, at the equilibrium geometry. For $F^{•}$, the 2s orbital (HOMO–1) at –30.5 eV is not shown. Computed at BLYP-D3(BJ)/TZ2P (continued overleaf).

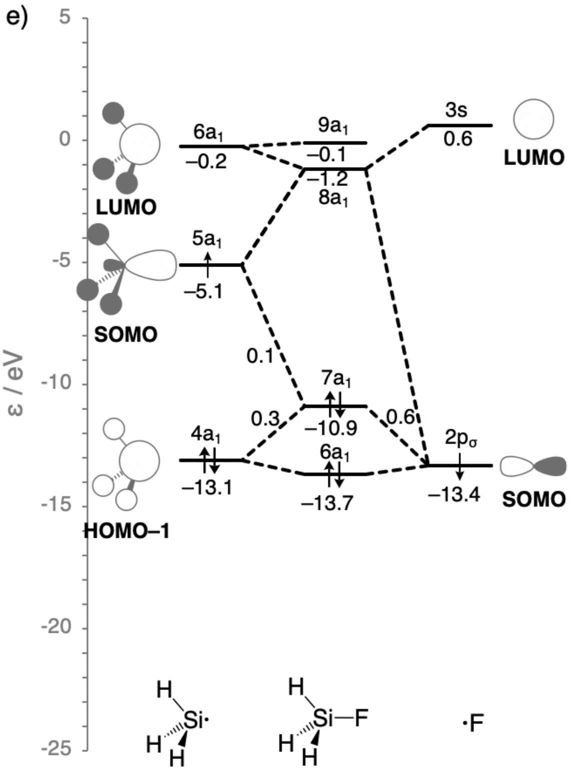

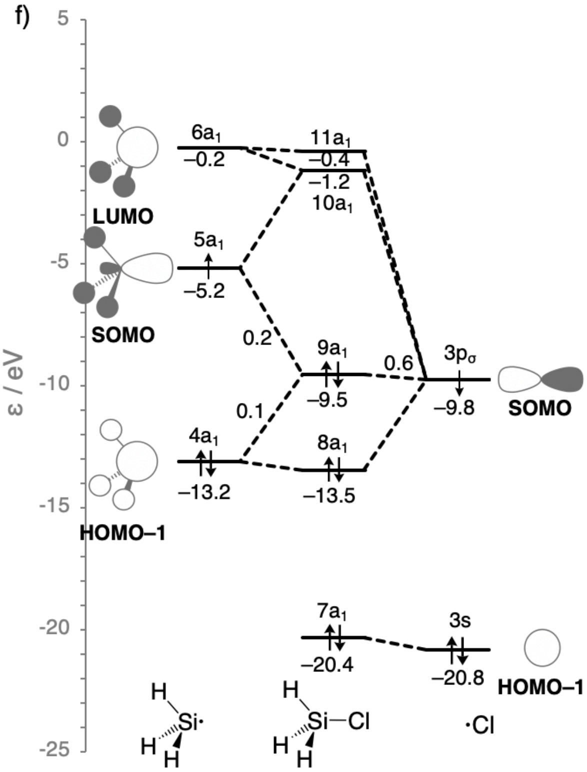

Figure S4. Continuation.

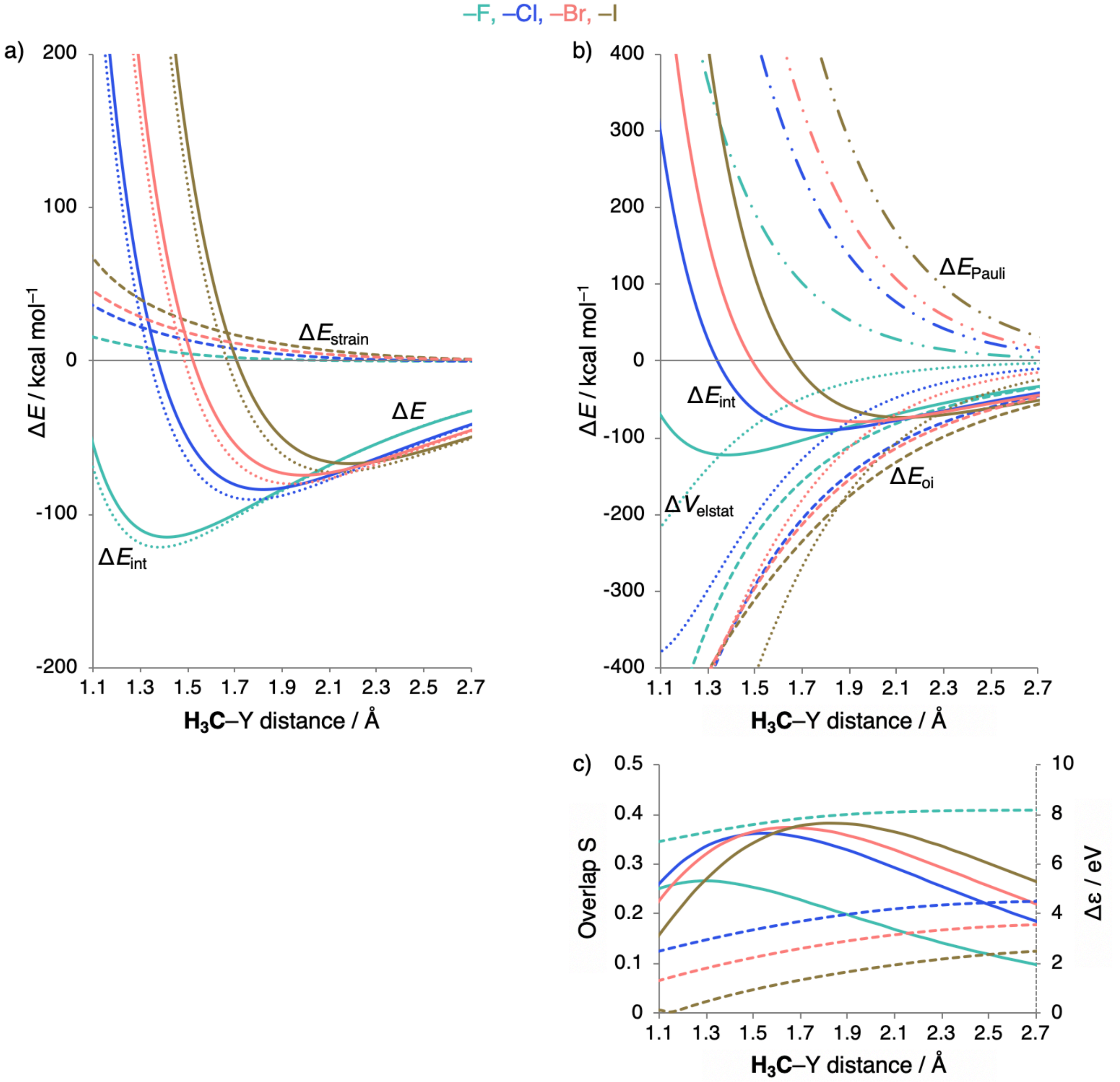

Figure S5. a) Activation strain analysis, b) energy decomposition analysis, and c) SOMO–SOMO overlap S and gap $\Delta\varepsilon$ as a function of the H_3C–Y distance (Y = F, Cl, Br, and I), computed at nonrelativistic BLYP-D3(BJ)/TZ2P.

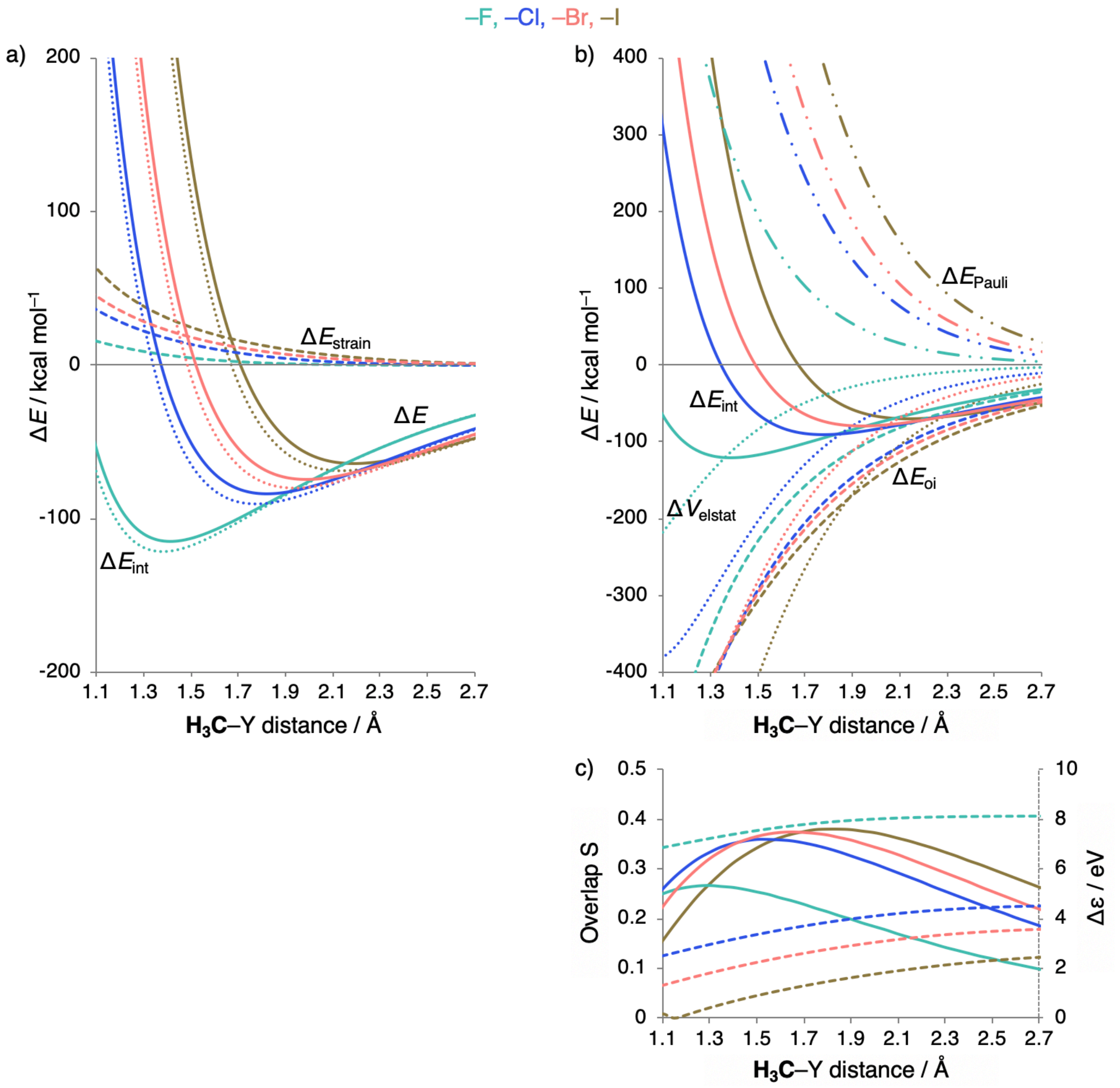

Figure S6. a) Activation strain analysis, b) energy decomposition analysis, and c) SOMO–SOMO overlap S and gap $\Delta\varepsilon$ as a function of the H_3C–Y distance (Y = F, Cl, Br, and I), computed at relativistic ZORA-BLYP-D3(BJ)/TZ2P.

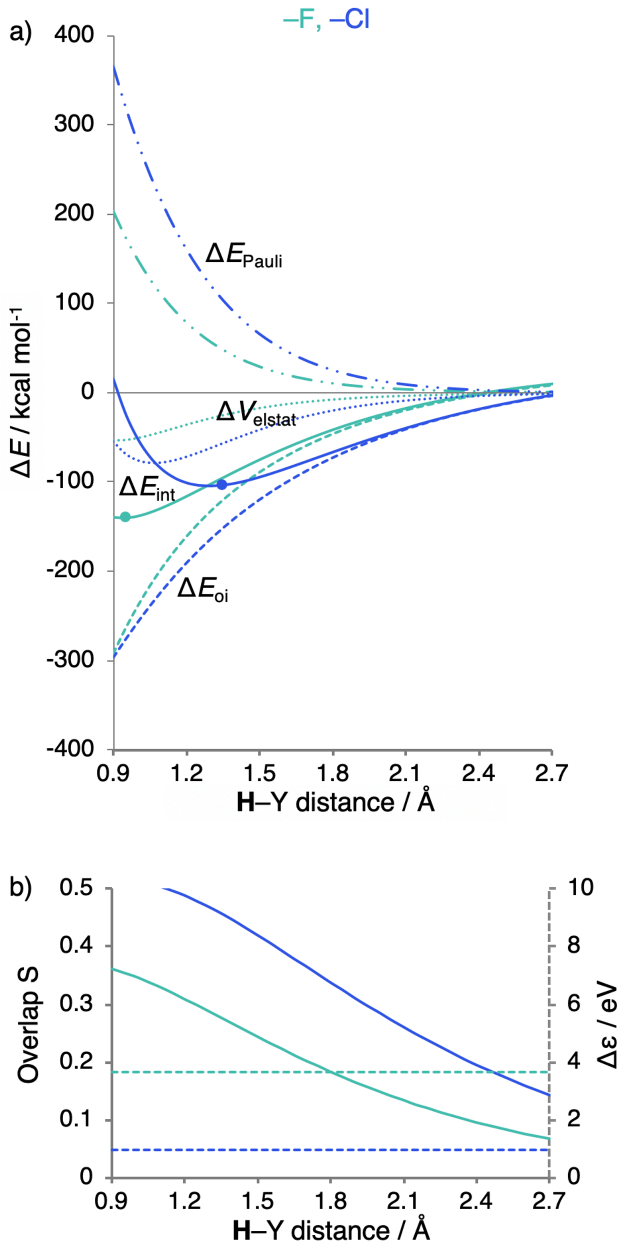

Figure S7. a) Energy decomposition analysis and b) SOMO–SOMO overlap S and gap $\Delta\varepsilon$ as a function of the H–Y distance (Y = F and Cl), computed at BLYP-D3(BJ)/TZ2P.

Table S4. Cartesian coordinates (in Å), ADF total bonding energies [TBE] (in kcal mol^{-1}), and number of imaginary frequencies NIMAG of the equilibrium geometries of all $H_nX–YH_n$ complexes in this study, computed at BLYP-D3(BJ)/TZ2P.

**$H_3C–CH_3$**	[−900.79]	NIMAG = 0	
C	0.000000	0.000000	0.769063
H	0.511179	0.885389	1.167098
H	0.511179	-0.885389	1.167098
H	-1.022359	0.000000	1.167098
C	0.000000	0.000000	-0.769063
H	-0.511179	0.885389	-1.167098
H	-0.511179	-0.885389	-1.167098
H	1.022359	0.000000	-1.167098

**$H_3C–NH_2$**	[−792.15]	NIMAG = 0	
C	0.242891	0.527498	0.000000
H	-0.637994	1.192756	0.000000
H	0.847508	0.765305	-0.883040
H	0.847508	0.765305	0.883040
N	-0.060117	-0.919503	0.000000
H	-0.619898	-1.165681	-0.817524
H	-0.619898	-1.165681	0.817524

**$H_3C–OH$**	[−672.63]	NIMAG = 0	
H	1.444108	0.704736	0.000000
O	1.090219	-0.200343	0.000000
H	-0.734876	0.386123	0.896669
C	-0.349843	-0.122608	0.000000
H	-0.734876	0.386123	-0.896669
H	-0.714733	-1.154030	0.000000

**$H_3C–F$**	[−535.15]	NIMAG = 0	
H	-0.519591	0.899958	-0.421649
H	-0.519591	-0.899958	-0.421649
H	1.039182	0.000000	-0.421649
C	0.000000	0.000000	-0.074107
F	0.000000	0.000000	1.339054

**$H_3C–SiH_3$**	[−800.12]	NIMAG = 0	
Si	0.000000	0.000000	0.920766
H	0.697312	1.207780	1.446516
H	0.697312	-1.207780	1.446516
H	-1.394624	0.000000	1.446516
C	0.000000	0.000000	-0.967545
H	-0.512426	0.887548	-1.357836
H	-0.512426	-0.887548	-1.357836
H	1.024852	0.000000	-1.357836

H_3C—PH_2 [−715.18] NIMAG = 0

C	0.234380	-0.708165	0.000000
H	-0.786533	-1.099302	0.000000
H	0.762169	-1.079058	0.885270
H	0.762169	-1.079058	-0.885270
P	0.316496	1.171950	0.000000
H	-0.644340	1.396817	1.034709
H	-0.644340	1.396817	-1.034709

H_3C—SH [−610.01] NIMAG = 0

H	1.577766	-1.086648	0.000000
S	1.378077	0.251838	0.000000
H	-0.845071	-0.299142	-0.898348
C	-0.467548	0.195403	0.000000
H	-0.845071	-0.299142	0.898348
H	-0.798152	1.237691	0.000000

H_3C—Cl [−495.06] NIMAG = 0

H	-0.518678	0.898377	-0.498734
H	-0.518678	-0.898377	-0.498734
H	1.037356	0.000000	-0.498734
C	0.000000	0.000000	-0.162122
Cl	0.000000	0.000000	1.658324

H_2N—NH_2 [−668.15] NIMAG = 0

H	0.413812	1.081866	-0.801060
N	-0.130960	0.744294	0.000000
H	0.413812	1.081866	0.801060
H	-0.413812	-1.081866	0.801060
N	0.130960	-0.744294	0.000000
H	-0.413812	-1.081866	-0.801060

H_2N—OH [−538.97] NIMAG = 0

H	-0.337969	-0.813073	-0.826262
N	-0.046137	-1.347627	0.000000
H	-0.337969	-0.813073	0.826262
O	1.420236	-1.349657	0.000000
H	1.714619	-0.416723	0.000000

H_2N—F [−393.71] NIMAG = 0

H	-0.518435	0.969550	0.816616
N	0.063087	0.732143	0.000000
H	-0.518435	0.969550	-0.816616
F	-0.063083	-0.732142	0.000000

H_2N—SiH_3 [−710.11] NIMAG = 0

Si	0.055160	-0.666997	0.000000
H	-1.303023	-1.295953	0.000000
H	0.762302	-1.133117	1.223201
H	0.762302	-1.133117	-1.223201

N	0.092995	1.069727	0.000000
H	-0.184868	1.579729	0.833256
H	-0.184868	1.579729	-0.833256

H_2N–PH_2 [–611.80] NIMAG = 0

H	0.600483	1.205607	-0.813776
N	0.097270	0.842160	0.000000
H	0.600483	1.205607	0.813776
H	-0.758454	-1.156278	1.025984
P	0.218672	-0.940818	0.000000
H	-0.758454	-1.156278	-1.025984

H_2N–SH [–499.38] NIMAG = 0

H	1.256136	-0.428202	-0.836274
N	0.864181	0.002343	0.000000
H	1.256136	-0.428202	0.836274
S	-0.864988	-0.004524	0.000000
H	-1.168455	-1.339575	0.000000

H_2N–Cl [–370.87] NIMAG = 0

H	0.632717	1.098317	-0.815680
N	0.041753	0.899269	0.000000
H	0.632717	1.098317	0.815680
Cl	-0.041755	-0.899267	0.000000

HO–OH [–403.29] NIMAG = 0

H	-0.972237	-0.723262	-0.268670
O	-0.744554	0.062012	0.268670
O	0.744554	-0.062012	0.268670
H	0.972237	0.723262	-0.268670

HO–F [–246.59] NIMAG = 0

O	0.735785	0.034898	0.000000
H	0.910085	-0.932698	0.000000
F	-0.735781	-0.034900	0.000000

HO–SiH_3 [–603.35] NIMAG = 0

Si	0.387639	0.116108	0.000000
H	0.913715	-0.584096	-1.207006
H	0.913715	-0.584096	1.207006
H	0.834847	1.529604	0.000000
O	-1.280627	0.179803	0.000000
H	-1.769288	-0.657324	0.000000

HO–PH_2 [–502.50] NIMAG = 0

H	0.301172	1.114643	1.027975
P	0.665363	0.184668	0.000000
H	0.301172	1.114643	-1.027975
O	-0.753258	-0.736119	0.000000
H	-0.514449	-1.677835	0.000000

HO—SH [−376.46] NIMAG = 0

H	0.951912	-0.536944	1.834155
S	0.372680	-0.681672	0.608858
O	1.791274	-0.892229	-0.314961
H	2.054932	-0.005693	-0.625077

HO—Cl [−239.19] NIMAG = 0

O	0.871566	-0.033410	0.000000
H	1.113064	0.915461	0.000000
Cl	-0.871566	0.033410	0.000000

F—F [−79.52] NIMAG = 0

F	0.000000	0.000000	-0.009642
F	0.000000	0.000000	1.429642

F—SiH$_3$ [−474.79] NIMAG = 0

Si	0.000000	0.000000	-0.046142
H	-0.704779	1.220712	-0.510855
H	-0.704779	-1.220712	-0.510855
H	1.409557	0.000000	-0.510855
F	0.000000	0.000000	1.578707

F—PH$_2$ [−368.45] NIMAG = 0

H	0.468009	0.529418	-1.025798
P	-0.508911	0.290977	0.000000
H	0.468009	0.529418	1.025798
F	-0.427107	-1.349813	0.000000

F—SH [−233.60] NIMAG = 0

H	-0.676671	-0.880622	0.000000
S	-0.493164	0.466836	0.000000
F	1.169835	0.413786	0.000000

F—Cl [−89.04] NIMAG = 0

F	0.000000	0.000000	-0.026256
Cl	0.000000	0.000000	1.656256

H$_3$Si—SiH$_3$ [−690.70] NIMAG = 0

Si	0.000000	0.000000	1.178149
H	0.698791	1.210341	1.695858
H	0.698791	-1.210341	1.695858
H	-1.397581	0.000000	1.695858
Si	0.000000	0.000000	-1.178222
H	-0.698804	-1.210364	-1.695834
H	1.397608	0.000000	-1.695834
H	-0.698804	1.210364	-1.695834

H$_3$Si—PH$_2$ [−616.56] NIMAG = 0

Si	-0.056628	-0.789671	0.000000

H	-1.542900	-0.878704	0.000000
H	0.469812	-1.476257	1.211245
H	0.469812	-1.476257	-1.211245
P	0.753491	1.349594	0.000000
H	-0.127582	1.783930	1.038531
H	-0.127582	1.783930	-1.038531

H_3Si–SH [–523.94] NIMAG = 0

Si	-0.500823	-0.197596	0.000000
H	-1.036855	0.481425	1.209636
H	-0.903641	-1.626484	0.000000
H	-1.036855	0.481425	-1.209636
S	1.666486	-0.242748	0.000000
H	1.811687	1.103977	0.000000

H_3Si–Cl [–420.27] NIMAG = 0

Si	0.000000	0.000000	-0.133814
H	-0.703168	1.217922	-0.604857
H	-0.703168	-1.217922	-0.604857
H	1.406335	0.000000	-0.604857
Cl	0.000000	0.000000	1.948386

H_2P–PH_2 [–535.33] NIMAG = 0

H	0.916769	-1.279216	1.027557
P	-0.069014	-1.138645	0.000000
H	0.916769	-1.279216	-1.027557
H	-0.916769	1.279216	-1.027557
P	0.069014	1.138645	0.000000
H	-0.916769	1.279216	1.027557

H_2P–SH [–435.65] NIMAG = 0

H	1.033297	0.799786	1.031302
P	0.810123	-0.163729	0.000000
H	1.033297	0.799786	-1.031302
S	-1.356144	-0.046593	0.000000
H	-1.520573	-1.389250	0.000000

H_2P–Cl [–322.91] NIMAG = 0

H	-0.790237	-0.675197	-1.025948
P	-0.034731	-1.327259	0.000000
H	-0.790237	-0.675197	1.025948
Cl	1.590744	0.021841	0.000000

HS–SH [–325.16] NIMAG = 0

H	0.912480	1.279191	0.473108
S	-0.035478	1.050303	-0.473108
S	0.035478	-1.050303	-0.473108
H	-0.912480	-1.279191	0.473108

HS–Cl [–201.91] NIMAG = 0

H	1.206408	1.323047	0.000000
S	1.041342	-0.023579	0.000000
Cl	-1.041305	0.023588	0.000000

Cl–Cl [–72.23] NIMAG = 0

Cl	0.000000	0.000000	-0.028857
Cl	0.000000	0.000000	2.018857

Table S5. Cartesian coordinates (in Å), ADF total bonding energies [TBE] (in kcal mol^{-1}), and number of imaginary frequencies NIMAG of the equilibrium geometries of all radical species in this study, computed at BLYP-D3(BJ)/TZ2P.

**$CH_3^•$**	[−404.37]	NIMAG = 0	
C	0.000000	0.000000	1.066517
H	0.541956	0.938695	1.066517
H	0.541956	-0.938695	1.066517
H	-1.083912	0.000000	1.066517

**$NH_2^•$**	[−300.48]	NIMAG = 0	
N	0.000000	0.000000	0.399196
H	-0.810269	0.000000	1.046744
H	0.810269	0.000000	1.046744

**$OH^•$**	[−173.14]	NIMAG = 0	
H	1.446840	0.711722	0.000000
O	1.087487	-0.207329	0.000000

**$F^•$**	[−15.51]	NIMAG = 0	
F	0.000000	0.000000	0.000000

**$SiH_3^•$**	[−308.22]	NIMAG = 0	
Si	0.000000	0.000000	0.779159
H	0.709576	1.229022	1.235391
H	0.709576	-1.229022	1.235391
H	-1.419152	0.000000	1.235391

**$PH_2^•$**	[−238.71]	NIMAG = 0	
P	0.000000	0.000000	-0.240066
H	1.025282	0.000000	0.762654
H	-1.025282	0.000000	0.762654

**$SH^•$**	[−130.63]	NIMAG = 0	
H	1.578118	-1.089004	0.000000
S	1.377725	0.254194	0.000000

**$Cl^•$**	[−6.54]	NIMAG = 0	
Cl	0.000000	0.000000	0.000000
